# Loss Factor Behavior of Thermally Aged Magnetorheological Elastomers

**DOI:** 10.3390/ma14174874

**Published:** 2021-08-27

**Authors:** Siti Aishah Abdul Aziz, Saiful Amri Mazlan, Ubaidillah Ubaidillah, Norzilawati Mohamad, Michal Sedlacik, Nur Azmah Nordin, Nurhazimah Nazmi

**Affiliations:** 1 Engineering Materials and Structures (eMast) iKohza, Malaysia-Japan International Institute of Technology (MJIIT), Universiti Teknologi Malaysia, Jalan Sultan Yahya Petra, Kuala Lumpur 54100, Malaysia; aishah118@gmail.com (S.A.A.A.); amri.kl@utm.my (S.A.M.); nurazmah.nordin@utm.my (N.A.N.); nurhazimah@utm.my (N.N.); 2 Department of Mechanical Engineering, Faculty of Engineering, Universitas Sebelas Maret, Surakarta 57126, Indonesia; ubaidillah_ft@staff.uns.ac.id; 3 Faculty of Engineering, Universiti Malaysia Sabah, Jalan UMS, Kota Kinabalu 88400, Malaysia; 4 Centre of Polymer Systems, University Institute, Tomas Bata University in Zlín, Trida T. Bati 5678, 760 01 Zlín, Czech Republic; msedlacik@utb.cz; 5 Department of Production Engineering, Faculty of Technology, Tomas Bata University in Zlín, Vavrečkova 275, 760 01 Zlín, Czech Republic

**Keywords:** magnetorheological elastomer, material elasticity, loss factor, damping properties, thermal aging, phase shift angle, rheological properties

## Abstract

Polymer composites have been widely used as damping materials in various applications due to the ability of reducing the vibrations. However, the environmental and surrounding thermal exposure towards polymer composites have affected their mechanical properties and lifecycle. Therefore, this paper presents the effect of material-temperature dependence on the loss factor and phase shift angle characteristics. Two types of unageing and aging silicone-rubber-based magnetorheological elastomer (SR-MRE) with different concentrations of carbonyl iron particles (CIPs), 30 and 60 wt%, are utilized in this study. The morphological, magnetic, and rheological properties related to the loss factor and phase shift angle are characterized using a low-vacuum scanning electron microscopy, and vibrating sample magnetometer and rheometer, respectively. The morphological analysis of SR-MRE consisting of 30 wt% CIPs revealed a smoother surface area when compared to 60 wt% CIPs after thermal aging due to the improvement of CIPs dispersion in the presence of heat. Nevertheless, the rheological analysis demonstrated inimitable rheological properties due to different in-rubber structures, shear deformation condition, as well as the influence of magnetic field. No significant changes of loss factor occurred at a low CIPs concentration, whilst the loss factor increased at a higher CIPs concentration. On that basis, it has been determined that the proposed changes of the polymer chain network due to the long-term temperature exposure of different concentrations of CIPs might explain the unique rheological properties of the unaged and aged SR-MRE.

## 1. Introduction

Mechanical vibrations, which are caused by oscillations in mechanical dynamic systems such as in manufacturing processes or in-home appliances are an unwanted phenomenon in our daily lives [1]. Moreover, these mechanical vibration forms can lead to excessive noise, which have a negative impact on labor safety, manufacturing efficiency, and productivity [2,3]. Often, these types of mechanical vibrations can cause minor or severe performance and safety issues in engineering systems [4]. To date, polymer composites or elastomers have been broadly used in various applications such as automotive [5,6], machinery products, and even in aviation industry [7,8,9] due to their viscoelasticity in order to lessen the vibration. Magnetorheological elastomer (MRE) is one type of polymer composite that can potentially be used not only to reduce but to achieve the desired vibration or as adjustable damping materials. Their tunable and reversible modulus as well as high stability in the presence of magnetic field make them a suitable material for the application of vibration absorbers. In fact, some tunable and reversible concepts of MREs have been reported previously, related to sensors [10,11,12], electromagnetic interference shielding [13,14], and vibration absorbers [15,16,17]. For example, Shabdin et al. [10] demonstrated that an MRE with graphite was capable of detecting force up to 100 N at 7 certain magnetic field strengths and exhibited controllable conductivity, which made this kind of material potential of use as a force sensor. Meanwhile, Sedlacik et al. [13] experimentally evaluated the performance of two different types of MREs consisting of silicone or thermoplastic matrix with different surface modifications of particles. The study revealed that the surface modification of particles influenced the electromagnetic shielding properties. On the other hand, Jeong et al. [17] designed an MRE-based adaptive tuned dynamic vibration absorber, where a vibration reduction of 8.38 dB has been achieved for the target frequency range of 110–130 Hz.

The MRE has been considered a functional material that can dissipate mechanical energy into heat [18]. Generally, the ability of an elastomer to dissipate energy can be determined by internal damping of either tan δ or loss factor. The requirement of tan δ > 0.3 over a broad temperature range needs to be achieved by high-performance damping materials [19,20]. In addition, it has been reported that the reliability and stability of the device or any system depends on its damping capacity, which could be affected by the composition of the polymer composite, MRE or rubber mixtures [21,22,23,24,25,26]. The damping property or tan δ is characterized as the energy dissipation of the vibration system or materials that can be presented by different parameters, such as loss factor and phase difference angle tangent. Damping occurs when an external oscillating force is applied by a mechanical energy dissipation, in which part of the mechanical energy is transformed into other types of energy, such as heat due to the internal friction of the molecular chain. For this purpose, the energy absorption capacity is reflected by the damping. Jong et al. [25] fabricated soy spent flakes with different concentrations of carbon black (CB) (10–30 wt%). The study revealed that the tan δ decreased with an increment of filler contents, where the filler was related to network structures that were responsible for heat dissipation. In another study, Joy et al. [27] examined the effect of adding different concentrations of hexagonal boron nitride (h-BN) nano powder (0.1 to 1 wt%) on polymer chain confinement, and thermal and mechanical properties of epoxy. The results showed that the height of tan δ peak decreased with the increasing h-BN concentration, which was related to the energy damping characteristics of the material. The increase in h-BN concentration prevented matrix chain motions in the nanocomposites. It is well understood that through internal and external vibrations, the structures should remain intact and undamaged in the engineering system. Nevertheless, the presence of temperature has some effects toward the general viscoelastic materials, as the stiffness, loss factor, and the damping properties have significantly decreased by increasing the temperature [19,28,29,30,31].

Some researchers have investigated the effect of temperature on mechanical and rheological properties of MREs. As such, Yunus et al. [30] studied the effect of different temperatures on epoxidized natural rubber (ENR) based MRE with different concentrations of carbonyl iron particles (CIPs) (up to 70 wt%). They found that the amount of CIPs certainly affects the thermal behavior of ENR-based MRE as the storage modulus increased with the increment of CIPs content and temperature. However, the effect of temperature on the loss factor has not been reported in their study. While Zhang et al. [32] investigated the effect of combination of silicone rubber (SR) with ethylene propylene diene monomer rubber (EPDM) for increasing the damping properties at a high temperature region. The results demonstrated that at 140 °C, the loss factor increased with the increment of EPDM contents due to the fact that the easy movement of the molecule as the material was more viscous at high temperature. Hence, the increment of EPDM led to the decrement of thermal stability. Later on, Qi et al. [31] prepared an EPDM/methylvinyl SR (MVQ) with CIPs surface silica coating in order to improve the thermostability and interfacial interaction properties. The results showed that the loss factor increased with increasing the EPDM content. In fact, the initial loss factor was also enhanced due to an interfacial defect caused by thermal aging, which resulted in a higher interfacial damping. Recently, Aziz et al. [33] studied the deterioration of rheological properties related to the phase shift angle (δ) of SR-based MRE with 60 wt% under the effect of thermal aging. The results demonstrated that the temperature has altered and simultaneously increased the phase shift angle (δ) along the strain, predominantly at a higher strain (>1%).

Pertaining to this situation, numerous studies have been investigated on the influence of temperature on the rheological and mechanical properties of polymer composites and MREs. Most of the studies have focused on improving the damping properties, while limited studies have looked at long-term exposure to temperature and aging properties toward the loss factor of MRE. Increasing the temperature could make these viscoelastic materials unable to adapt to certain specific operating conditions, such as alternating extreme processing of high and low temperatures or prolonged exposure to heat. In fact, the effect of thermal energy causing the ageing of MREs and its correlation with magnetic particles concentration is still unknown. As previous studies have showed that the damping characteristics of viscoelastic material were sensitive to temperature, therefore, a comprehensive study is needed to investigate the materials performance in a long-term treatment. Relatively, the aim of this study is to examine the morphological, magnetic, and rheological properties of unaged and aged SR-MREs with different concentrations of CIPs, specifically on the loss factor and its relation to the phase shift angle. Hence, this study provides fundamental knowledge on the rheological properties change within the low and high strain deformation for thermally aged MRE at different CIPs concentrations. On the other hand, it provides a critical assessment of the development of material quality and performance.

## 2. Results and Discussion

### 2.1. Morphological Analysis

**Figure 1:**
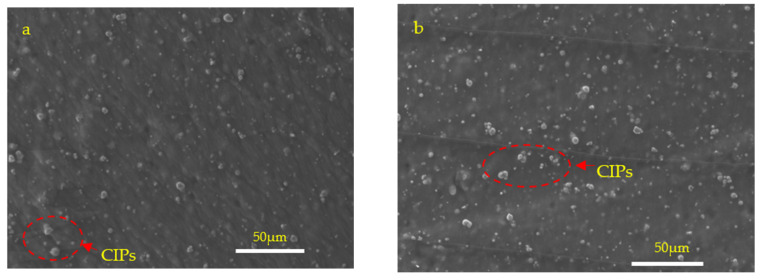

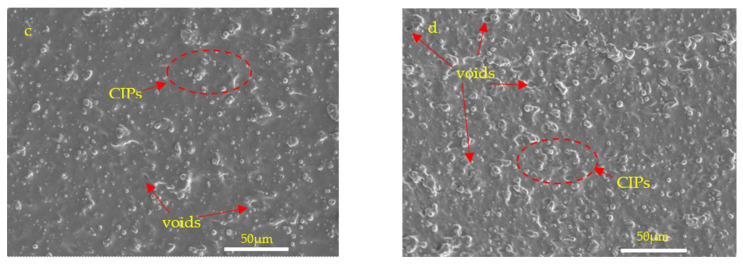
Morphological analysis of the SR-MREs cross-section with 30 wt% CIPs (**a**) before and (**b**) after thermal aging, and SR-MREs with 60 wt% (**c**) before and (**d**) after thermal aging.

The morphological observation of SR-MRE samples with different concentrations of CIPs before and after thermal aging are shown in Figure 1. As depicted in Figure 1a, the homogeneous distribution with random orientation of 30 wt% CIPs embedded in the SR-MRE before thermal aging is captured. Meanwhile, due to the thermal aging process (Figure 1b), the SR-MRE surface is visibly smoother than before thermal aging. Nonetheless, CIPs were well dispersed in the matrix with few small agglomerations after thermal aging.

In addition, the morphological analyses for the SR-MRE with 60 wt% of CIPs are shown in Figure 1c,d. Apparently, as can be seen in Figure 1c, the increase in CIPs concentration has contributed to a rougher surface on unaged SR-MRE sample with 60 wt% of CIPs compared to 30 wt% of CIPs (Figure 1a). In addition, more CIPs agglomerations with some voids have also been observed. Nevertheless, the aged SR-MRE sample with 60 wt% of CIPs (Figure 1d) depicted an even more rougher surface, CIPs agglomerations, and voids in comparison to the unaged sample (Figure 1c). On the other hand, the unaged samples in Figure 1a,c have smoother cross-section surfaces, where the distribution of CIPs was better than for aged samples in Figure 1b,d. In fact, rougher surfaces in Figure 1d have been observed due to the higher embedded CIPs contents in the SR-MRE in comparison to Figure 1a,b. 

It is known that the solid/liquid transformation process occurs during the thermal treatment or heat exposure, as well as the mutual thermophysical properties between both the material and mold. This process may affect the changes of morphological behavior inside the SR-MRE samples. It can be assumed, by comparing both morphological analyses, that the heat path during the thermal aging process has changed the microstructure of the SR-MREs. Thus, the thermal might improve or deteriorate the dispersion of the CIPs inside the SR-MRE. Generally, in pure SR, during exposure to heat, the molecules vibrate faster, thus weakening the intermolecular forces of polymer chains and resulting in expanding the dimension. At the same time, the polymer chains stretch under the influence of heat, where the SR softens and increases in material elasticity. Similarly, the expansion of rubber in the composite or MRE samples was influenced by thermal aging even with the presence of filler. From the results obtained in Figure 1b,d, the heat has altered the morphology including the interparticle interactions of CIPs inside the SR-MRE samples. This phenomenon can be explained due to the interchange of various microscopic forces exerted on the matrix and CIPs, which generally influence the matrix-particles and particle-particles interaction [34]. As stress accelerated the process due to continuous exposure to heat, the higher concentration of CIPs increased the stress concentration between the matrix-particles resulting in some agglomerations due to the weak compatibility between matrix-particles. In this condition, some voids might occur due to debonding of matrix-particles [35]. Meanwhile, at several areas, the particles seem to ‘extend’ further out by the softened matrix deformation due to the continuous heat exposure during thermal aging. Due to the continuous heat exposure, the polymer chain segment is becoming less efficient and thus, the usual orientation of the network chain was distracted due to the excessive cross-linking density [36]. Moreover, the presence of high concentration of CIPs hindered the rubber molecular chains as the CIPs gain more kinetic energy and vibrate faster with the increased temperature. During this treatment, the CIPs might ‘bump’ into each other thus, resulting in more CIPs agglomerations with some extended “stretchmarks”, which were observed especially after thermal aging.

### 2.2. Magnetic Properties

**Figure 2:**
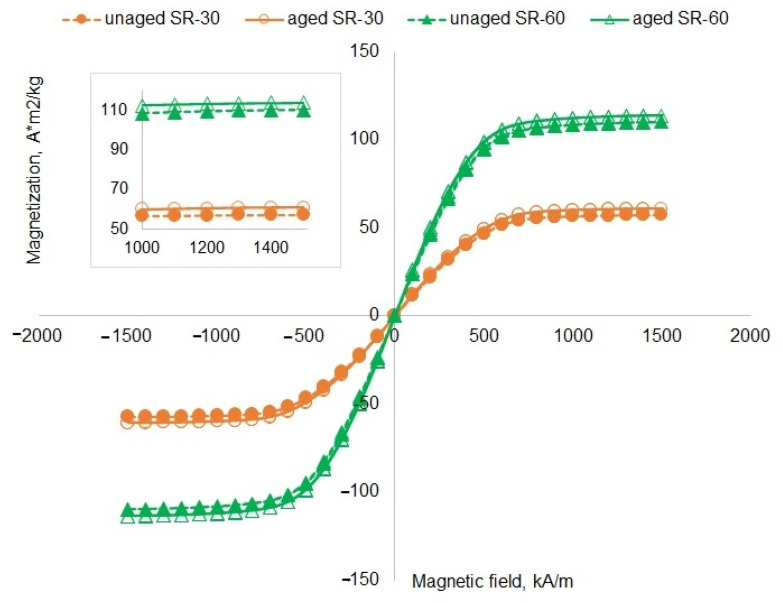
The magnetic properties of unaged and aged SR-MRE samples with different concentrations of CIPs.

The magnetic properties of unaged and aged SR-MRE samples are shown in Figure 2. It is well-known that the higher the CIPs concentration, the higher the magnetic saturation. Thus, the results depicted that SR-MRE with 60 wt% of CIPs (unaged and aged), has higher magnetic saturations as compared to SR-MRE with 30 wt% of CIPs. The summary of three sets of magnetic properties data with standard deviations is listed in Table 1.

Table 1 shows that the magnetic saturations (*M*_s_) for unaged and aged SR-MRE samples with 30 wt% of CIPs are 57.3 and 60.7 Am^2^/kg, while for 60 wt% of CIPs are 110 and 114 Am^2^/kg, respectively. The differences in *M*_s_ between unaged and aged SR-MRE samples with 30 and 60 wt% of CIPs was about 6 and 4%, respectively. It can be noted that the values of *M*_s_ were higher for both aged samples. The increase in *M*_s_ for both aged SR-MRE samples might be due to the uniform dispersion during the thermal aging process, which is in good agreement with the morphology analysis discussed vide supra (Figure 1). During the thermal aging procedure, the CIPs have gained thermal energy that caused the ‘vibration’ of particles which led to easy rotation of CIPs along the direction of magnetic field [37], thus, slightly changing their magnetization behavior. Meanwhile, the retentivity (*M*_R_) of both aged SR-MRE samples demonstrated an increasing trend after the thermal aging procedure indicating that the magnetic isotropy strength has increased with the influence of heat. Whilst the coercivity (*H*_c_) of the SR-MRE samples increased owing to the random orientation of ferromagnetic particles after thermal aging.

### 2.3. Rheological Properties 

#### 2.3.1. Loss Factor (tan δ) of SR-MRE Samples

**Figure 3:**
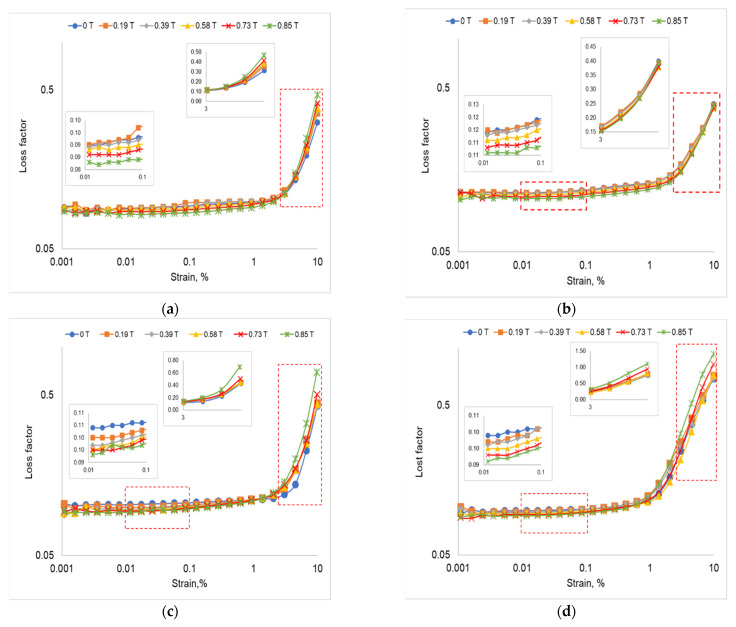
The loss factor of SR-MRE samples with 30 wt% of CIPs (**a**) before and (**b**) after thermal aging, and SR-MRE samples with 60 wt% of CIPs (**c**) before and (**d**) after thermal aging under various magnetic flux densities.

The loss factor of unaged and aged SR-MRE samples with different compositions of 30 and 60 wt% of CIPs is demonstrated in Figure 3. 

As shown in Figure 3, insignificant changes of loss factor are observed for all unaged and aged SR-MRE samples at a low strain (<1%). However, with the increment of strain (>1%), the loss factor of all SR-MRE samples was sharply increased. In another observation, the effect of magnetic flux density could be seen in two phases: At strain <1% and at strain >1%. As depicted in the enlarged figures for all conditions (Figure 3a–d), at strain <1%, the loss factor was higher at a lower magnetic flux density. However, the trend was in an opposite direction at strain >1%, in which the loss factor was higher at a higher magnetic flux density. In other words, initially, the SR-MRE samples demonstrated a small increment of loss factor with increasing strain until it reached 1%. Then, a rapid increase in loss factor has been obtained with a further increase in strain. On the contrary, the loss factor of SR-MRE samples has shown a unique characteristic by increasing the magnetic flux density along with increasing the strain. A higher loss factor has occurred at a lower magnetic flux density with an almost consistent gap at each applied current value at strain <1%. After strain >1%, this gap started to decrease until the value was overlapping to create a new trend, where eventually, a higher loss factor at a higher magnetic flux density has been observed.

In general, a low value of loss factor at strain <1% depicted the ability of the materials to store more energy/load rather than dissipating it. Then, at strain >1%, the materials were capable of dissipating energy attributed to strain softening of the polymer chains. This effect was due to the breakup of the matrix-filler and filler-filler network, which is also known as Payne effect. On the other hand, this Payne effect resulted in the interfacial friction between the matrix-filler and filler-filler caused by their agglomerations as well as their entanglements under continuous deformation. Moreover, under a large deformation, the complete chains between the matrix-filler and filler-filler might be abolished before rearranging again to the initial order. This behavior was believed to be the primary energy dissipation mechanism for such high loss factor. 

As demonstrated in Figure 3, in terms of the influence of magnetic flux density, the loss factor exhibits two characteristic regions (up and drop) along the increase of strain. The first decreasing region of loss factor corresponded to strain <1%, where the loss factor was slightly changed with the increment of magnetic flux density. The decreasing trend of loss factor was experienced by all SR-MRE samples that might be due to the occurrence of more chain links between SR and CIPs interfaces, and therefore, limited the CIPs mobility. Moreover, at a small deformation (strain <1%), the CIPs might be able to form a continuous phase through the SR matrix, thus, restricting the microscopic movement inside the MRE samples even with the influence of magnetic field. Meanwhile, for the onset strain for the second increasing region, the loss factor increased sharply with the increased magnetic flux density, particularly at a high deformation (strain >1%). It has to be mentioned that the filler-filler distance became incredibly convoluted under the action of the applied current after rupture of the matrix-filler and filler-filler networks due to the higher elasticity of SR-MRE [38]. Due to this phenomenon, the matrix-particle network was getting hard to move together along with the increment of the current and ultimately increased the friction between them. Eventually, the SR-MRE samples had more energy dissipation potential, hence increased in loss factor which agreed well with the micrograph analysis (Figure 1). 

**Figure 4:**
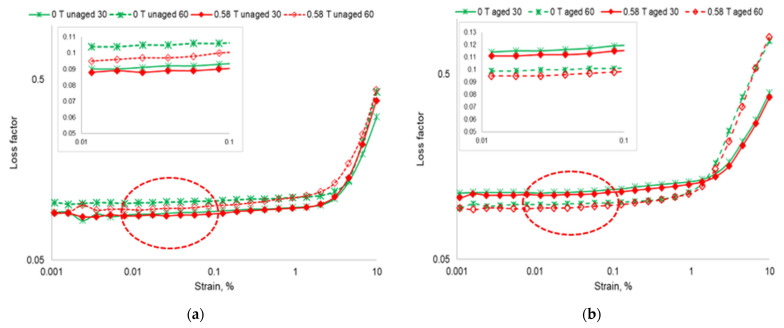
Comparison of loss factor of (**a**) unaged SR-MRE samples with 30 and 60 wt% of CIPs and (**b**) aged SR-MRE samples with 30 and 60 wt% of CIPs.

In comparison, the unaged and aged SR-MRE samples with different compositions (30 and 60 wt% of CIPs) at the off-state condition (0T) and on-state condition (0.58T) are demonstrated in Figure 4. Some changes of the loss factor were observed at all strains and magnetic flux densities for both unaged and aged samples.

As demonstrated in Figure 4, the loss factor increased with the increment of strain and concentration of CIPs. Moreover, the loss factor has also increased with the increment of the current applied. The increase in loss factor for unaged SR-MRE samples at a higher concentration of CIPs could achieve around 50% higher value in comparison to a lower CIPs concentration. Nevertheless, a unique trend of SR-MRE samples aging was observed, where the loss factor has dramatically increased with the strain increasing (>1%) and magnetic flux density. As such, the loss factor demonstrated a tremendous 1.8 times higher increase for SR-MRE based on 60 wt% of CIPs as compared to the 30 wt% one. It has been reported elsewhere that the different concentrations of filler have altered the loss factor behavior of the materials [39,40,41]. A progressive increase in loss factor could also be observed in both unaged and aged SR-MRE samples with a higher concentration of CIPs. An abrupt increase in loss factor was obtained in aged samples, particularly with a high CIPs concentration owing to the rise of interparticle interaction. Furthermore, due to the exposure to a high temperature during thermal aging, the viscosity of the SR-MRE was increased, thus, significantly restricted the movement of the polymer chains and CIPs structures. The extended contact between their long molecular chains led to enhanced intermolecular attractions that contributed to a resistance to flow. Owing to this, the polymer chains were more tangled, hence, contributed to a higher chance of generating voids, which is consistent with the morphological result (Figure 1d).

**Figure 5:**
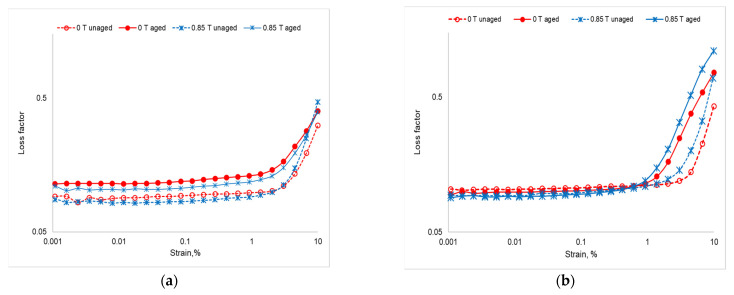
Comparison of loss factor of unaged and aged (**a**) SR-MRE samples with 30 wt% of CIPs and (**b**) SR-MRE samples with 60 wt% of CIPs.

Meanwhile, Figure 5 shows the comparison of loss factor of both unaged and aged SR-MRE samples with 30 and 60 wt% of CIPs at the off-state condition (0T) and on-state condition (0.85T). 

As shown in Figure 5a, the loss factor of unaged SR-MRE with 30 wt% of CIPs has lower values when a higher value of magnetic flux density is applied in comparison to the situation presented in Figure 4, which might be due to the interaction of the matrix-filler and filler-filler distribution inside the MRE. At a higher magnetic flux density, the filler-filler network tended to aggregate more owing to the rotation of magnetic moment in order to align along with an external magnetic field direction [42]. The aggregation inhibited a formation of continuous phase and decreased the dissipation energy, hence resulting in lower values of loss factor. However, the pattern was somewhat counterintuitive at strain >1% as the loss factor at a higher magnetic flux density tended to close the gap and eventually overcome the loss factor values at a lower magnetic flux density. The obtained results could be attributed to the fact that changes in the interaction of matrix-filler as well as the adhesion between these two phases occurred due to the increment of deformation [43]. Furthermore, a higher magnetic flux density could result in facilitating more frictions between the different phases of MRE, which partly explained the higher values of loss factor of the unaged SR-MRE with 30 wt% of CIPs. In contrast, the loss factor was slightly higher for aged SR-MRE with 30 wt% of CIPs compared to the unaged one at all strains and applied current. For the aged SR-MRE samples, continuous exposure to heat might result in an adsorption of polymer chain matrix onto the CIPs surface due to the excessive cross-link density. Therefore, the adsorption of polymer chain matrix could affect the dispersion degree of CIPs in the SR matrix and consequently the rheological properties, particularly the loss factor of the aged SR-MRE sample with 30 wt% of CIPs [44]. On the other hand, due to the thermal aging treatment and small shear deformation, the SR-MRE samples tended to have stronger magnetic particles interactions owing to a high elastic modulus at an elastic region. 

**Figure 6:**
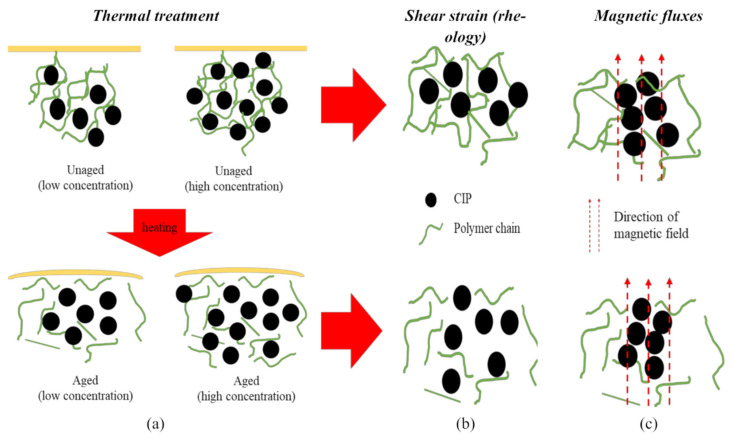
Matrix-filler interactions in unaged and aged SR-MRE samples under different external stimuli.

Meanwhile, a different phenomenon was observed for SR-MRE with 60 wt% of CIPs, which can be seen in Figure 5b. Some small changes of loss factor were observed at strain <1%. This trend was likely to be related to a higher concentration of CIPs, where more matrix-filler interactions could occur causing a decrease in mobility of the molecular chains surrounding the matrix interface. However, at strain >1%, the loss factor increased dramatically with the increment of strain. Furthermore, the values of loss factor were higher at a higher magnetic flux density. The increment of strain and magnetic flux density might lead to the occurrence of a micro-particle structure, in which the segment motion offered by the CIPs is hindered leading to the increment of loss factor at the filler-matrix interface [45]. Previously, as shown in Figure 1d, the interface changes occurred during the thermal aging as these chains have the ability to move towards each other [46]. Thus, the continuous exposure to the elevated temperature has increased the molecular chain mobility, which resulted in the reduction of force values to deform the SR-MRE sample. In the meantime, some appearances of void and ‘stretchmark’ in the SR-MRE helped the movement of CIPs and reduced the loss factor. The possible suggested phenomena of the matrix-filler interaction in the SR-MRE samples by the factors of thermal, shear strain, and magnetic fluxes are illustrated in Figure 6.

Figure 6a shows the matrix-filler interactions in SR-MRE samples before and after thermal aging. Unaged SR-MRE exhibited a compact structure by having many entanglement polymers chains that represented the matrix-filler interactions. Whilst, after thermal aging, the heat weakens the matrix-filler interactions by breaking the polymer chains and simultaneously offering some spaces for particles movement [47]. This illustration supports the hypothesis that the heat treatment exerted during the thermal process played a foremost role in affecting the movement behavior of CIPs as well as the matrix-filler interaction inside the SR-MREs. Generally, the particles are tightly packed together in a solid phase. In this treatment, the particles are strongly adhered together to allow any movement from place to place, but instead, the particles vibrate on their position in the structure. With the influence of elevated temperature, the particles gain kinetic energy and vibrate faster and zealous. However, the interparticle interactions strongly depend on the distance between the neighboring particles. Therefore, at a low concentration of CIPs, the repulsive force dominated over the attractive one resulting in a uniform distribution of particles inside SR-MRE. Nonetheless, a closer distance between the particles at a high concentration of CIPs contributed to a stronger attractive force and dominated over the repulsive force, hence leading to CIPs aggregations. These results agreed well with the previous study [48].

Figure 6b shows a displacement of matrix and CIPs in the SR-MRE during the shear deformation. The polymer chain samples tended to stretch in the direction of the shear deformation. For the unaged sample, a reduction in polymer chain entanglements was observed in responding to strain deformation. On the other hand, for the aged SR-MRE, due to a locally soft network, the polymer chains have more lateral degrees of freedom, hence some movements of CIPs were observed during the continuous shear deformation. In addition, the deformation could be divided into two conditions, where the first condition was referred to a low strain, while the second condition referred to a high strain. At a low strain, the elasticity of matrix-filler is capable of maintaining the unstretched structure due to the small deformation angle. However, at a high strain, the polymer chains stretched further, thus tending to break-out as the timescale for the relaxation process seemed to decrease with the increased shear applied. In the meantime, the extended chains configuration ‘drags’ along the CIPs, thus leaving some small spaces known as a void inside the SR-MRE. Nonetheless, in the presence of a magnetic field as shown in Figure 6c, the magnetic dipole of the CIPs interacted with the magnetic field and aligned in the same direction. As the external magnetic field increased, a stronger interaction between CIPs has been obtained. As a result, the CIPs tended to move and formed a chain-like structure parallel to the direction of magnetic field. It was noted that in a low concentration of CIPs, a longer distance between them created a higher loss factor of the MRE. Thus, the CIPs were allowed to vibrate freely concerning their equilibrium positions, where the loss factor tended to increase parallel with the magnetic field’s increment. 

#### 2.3.2. Phase Shift Angle (δ)

**Figure 7:**
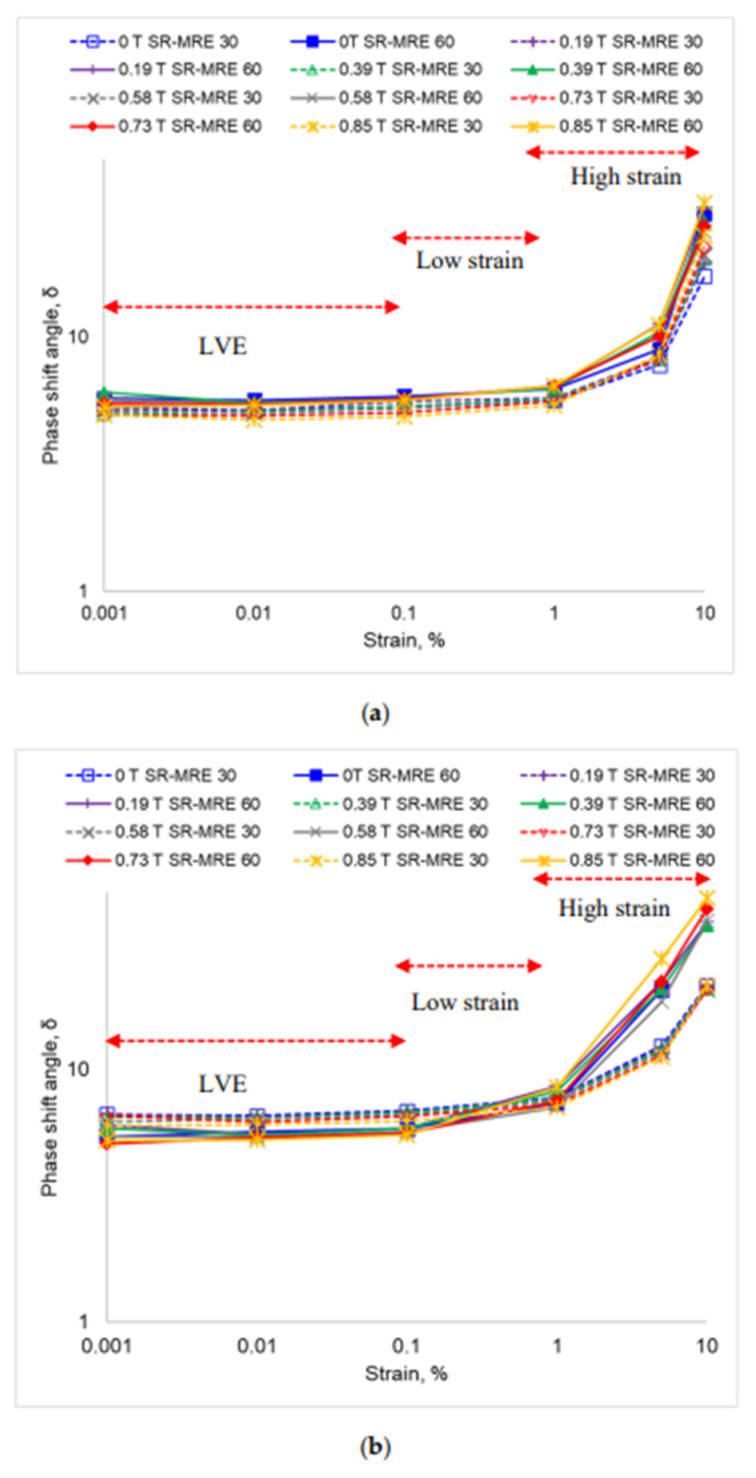
Comparison of phase shift angle of both (**a**) unaged and (**b**) aged SR-MRE samples at different strains and magnetic fields strength.

Generally, the δ represents the phase shift angle that occurred between the stress (force from external magnetic field) and strain (deformation). Figure 7 depicts the comparison of phase shift angle of unaged and aged SR-MRE samples with different compositions (30 and 60 wt% of CIPs). As shown in Figure 7a, all unaged SR-MRE samples demonstrated an increasing trend of phase shift angle with the increment of magnetic field applied. The higher CIPs concentration in the SR-MRE displayed higher phase shift angles by almost 100% increment as compared to the lower CIPs concentration. In the meantime, a constant phase shift angle for both SR-MRE samples was obtained within the LVE region (up to 0.1%). However, at the low strain region (0.1–1%), some noticeable change was observed for both SR-MRE samples in which the phase shift angle tended to increase with the increment of magnetic field applied. Then, the phase shift angle dramatically increased at high strain region (1–10%). In addition to the effect of shear, the phase shift angle was also increased with the increment of the magnetic field. Within the LVE region, no differences were observed due to the strong matrix-filler interactions and high elasticity of SR-MRE. As the strain slightly increased in the low strain region, the phase shift angle was slightly larger with the increment of CIPs concentration or magnetic field applied. Thus, these two parameters may affect the viscoelasticity of the SR-MRE due to the CIPs movement and changes in the matrix-filler interactions. With a further increase in the strain, obviously larger values of the phase shift angle were obtained due to the CIPs agglomeration, which decreased the elasticity of SR-MRE. This result is supported with the morphological analysis in Figure 1.

Meanwhile, some changes were observed for the aged SR-MRE samples with 30 and 60 wt% of CIPs at all strains, as shown in Figure 7b. The phase shift angle increased for both samples immensely at high strain region. For the SR-MRE samples with 30 wt% of CIPs, an interesting finding of a constant value of the phase shift angle with the increment of magnetic field was observed. This result might be attributed to the binding and network connection of matrix-matrix and matrix-filler that retained the SR-MRE system’s essential elastic nature. Hence, providing an additional elasticity and the ability to restore deformation. Nonetheless, for the SR-MRE with 60 wt% of CIPs, the phase shift angle was always higher than for the SR-MRE with 30 wt% of CIPs at all strains due to a higher concentration of CIPs. Furthermore, a noticeable increase in the phase shift angle of SR-MRE with 60 wt% of CIPs was detected at a higher strain region (>1%). The changes in the phase shift angle were caused by the presence of magnetic field strength (force), which resulted in some delay in the replacement reaction due to the time taken to reorient the matrix-filler linkages under deformation. 

Within the LVE region, referring to the long-time scales, the reorientation of polymer chains was very extensive. Therefore, the motion of polymer chains was able to synchronize with the external force, thus resulting in a small rubbery plateau with nearly constant value of loss factor. Meanwhile, at the low strain region, the polymer chains and CIPs orientation due to some movements of polymer chains might experience some delays in keeping pace with the alternating stress and forces. Consequently, energy dissipation occurred, and the material behaved as a viscoelastic manner. Nevertheless, at the high strain region, the phase shift angle increased dramatically for both SR-MRE samples. At high strain deformation, the material experienced a short relaxation time, therefore the polymer chains and CIPs might experience an inadequate time for reorientation. In the meantime, no coordination of the motion of the polymer chains with the external forces happened, resulting in a high friction. Additionally, the increase in phase shift angle might be due to the void’s appearance and more CIPs agglomeration, leaving a stretched network during the thermal aging as can be clearly seen in Figure 1d, resulting from the CIPs movement restriction. In addition to the polymer chains movement, the CIPs might also be dragged during the deformation, thus producing friction and generating heat. Moreover, the particles had smaller gaps between them due to a higher content of CIPs, therefore, vibrating in the presence of a magnetic field. The vibration increased with the increment of the magnetic field strength, resulting in more frictions among the particles, particularly at the agglomerated particles. However, the particles experienced a difficult ‘movement’ leading to more frictions due to the instantaneous dynamic interaction and the occurrence of a stretched network with some deep voids. In the meantime, the loss of interfacial contact due to the severe oscillation during shear deformation led to vibro-impact motions and non-linear dynamics patterns. 

## 3. Methodology

### 3.1. Samples Preparation

**Scheme 1:**
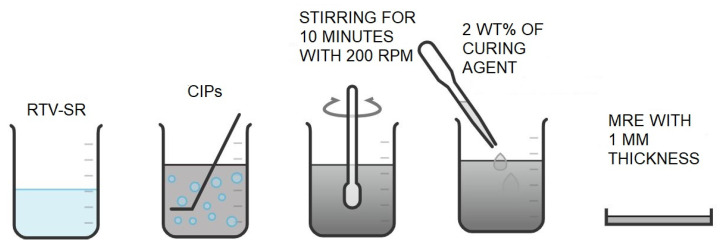
SR-MRE samples preparation.

Two different concentrations of CIPs, 30 and 60 wt%, at room temperature vulcanization (RTV) silicone based MRE (SR-MRE) samples were prepared in this study. Hence, the MRE samples consisted of spherical shapes of magnetic CIPs (OM grade, BASF, Germany) with an average size of 5 µm. The preparation of SR-MRE samples is illustrated in Scheme 1. 

The room temperature vulcanization (RTV) SR, NS 625 A (Nippon Steel Co., Tokyo, Japan) and CIPs were mixed for 10 min under 200 rotations per minute (rpm) at room temperature (25 °C) using a mechanical stirrer until a visually homogenous mixture was obtained. Additionally, 2 wt% of curing agent, NS 625 B (Nippon Steel Co., Tokyo, Japan) from the SR total was added into the SR-MRE samples and the system was continuously stirred for 1 min. Subsequently, the SR-MRE samples were transferred into a mold with a thickness of 1 mm and left for 2 h to complete the curing process at room temperature.

### 3.2. Samples Characterization 

In order to examine the thermal aging for both SR-MRE samples, an oven chamber (Carbolite Chamber Furnace, CW 1100, Hope, Derbyshire, UK) was utilized. The SR-MRE samples were constantly exposed to 100 °C for 72 h to obtain the aged samples. Then, the samples were left overnight at room temperature of 25 °C before the morphological analysis as well as the mechanical and rheological tests were conducted.

A low-vacuum scanning electron microscopy (LV-SEM, JEOL JSM-IT300, Japan) using a voltage of 5 kV, with a magnification of 500× and 3000× was used to examine the SR-MRE samples cross-section. The platinum layer was used to coat the samples cross-section to avoid any sample charging during performing a morphological observation of SR-MREs. Meanwhile, the magnetic properties of those SR-MREs before and after thermal aging were examined using the vibrating sample magnetometry (VSM, Microsense, FCM-10, Lowell, MA, USA). The samples were continuously vibrated during the experiments under varying magnetic fields from −1500 to 1500 kA/m. A modular compact rheometer equipped with a magnetorheology cell 70/1T MRD (MCR 302, Anton Paar, Graz, Austria) was used to investigate the rheological properties of SR-MREs samples, particularly the sweep strain under an oscillation mode at room temperature. During the rheological test, the magnetic field value that was applied to the samples was controlled by adjusting the applied currents with intervals of 0, 1, 2, 3, 4, and 5 A. By controlling the applied current value (0–5A), a respective magnetic field is generated by the coil, and the magnetic flux density of the samples was measured using a Teslameter that was attached to the device (refer to Table 2). Three sets of data for each test were recorded to maintain the consistency of the result obtained. As for the sweep strain test, variable strains from 0.001 to 10% with a constant frequency of 1 Hz were utilized.

## 4. Conclusions

The present study demonstrated the changes of loss factor and phase shift angle at linear, low, and high region deformation of unaged and aged SR-MRE samples at different CIPs concentrations. The morphology, magnetic, and rheological properties changes of these SR-MRE samples were thoroughly examined. For the aged SR-MRE sample at low concentration of CIPs, smooth and homogenous embedded CIPs in the sample have been observed. While, after thermal aging of the SR-MRE sample with a high concentration of CIPs, a rougher surface with more voids and agglomerations of CIPs was demonstrated. On the other hand, the thermal effect of aging process has increased the magnetic saturation of the aged sample to almost 100% as compared to the unaged SR-MRE samples. It is interesting to note that the loss factor was affected by the concentration of CIPs and magnetic field strength applied due to the changes of the matrix-filler and filler-filler interaction and orientation. The values of loss factor and phase shift angle were increased with the increment of the strain and magnetic field. The occurrence of a stretched network with some deep voids at a high CIPs concentration was due to the instantaneous dynamic interaction, where the particles experienced ‘movement’ obstruction that led to more frictions. This finding is very fascinating as different concentrations of CIPs exhibited complex rheological properties and morphological structures, particularly the loss factor and the phase shift angle in the presence of the long-term elevated temperature exposure.

## Figures and Tables

**Table 1:** The summary of magnetic properties of unaged and aged SR-MRE samples (30 and 60 wt% of CIPs) samples.

Sample	*M*_s_ (Am^2^/kg)	*M*_R_ (Am^2^/kg)	*H*_c_ (kA/m)
Unaged SR-MRE 30 wt%	57.3 ± 0.2	0.11 ± 0.002	9.78 ± 0.08
Aged SR-MRE 30 wt%	60.7 ± 0.3	0.12 ± 0.001	9.87 ± 0.04
Unaged SR-MRE 60 wt%	110.0 ± 0.5	0.13 ± 0.001	5.52 ± 0.01
Aged SR-MRE 60 wt%	114.6 ± 0.8	0.15 ± 0.002	5.64 ± 0.01

**Table 2:** The relationship between the applied current and magnetic flux density during the rheological test.

Applied Current (A)	Magnetic Flux Density (T)
0	0.00
1	0.19
2	0.39
3	0.58
4	0.73
5	0.85

## Data Availability

The data presented in this study are not publicly available at this time due to the data is also form part of an ongoing study.
